# Coping Styles and Defense Mechanisms in Healthy Young Adults—Correlations with tPA-BDNF Pathway

**DOI:** 10.3390/brainsci15060575

**Published:** 2025-05-26

**Authors:** Julia Pilecka, Jedrzej Wojciechowski, Weronika Bargiel, Maria Terczynska, Przemyslaw Zakowicz, Dawid Bojarski, Karolina Wasicka-Przewozna, Maria Skibinska

**Affiliations:** 1 The Student Scientific Society of Poznan University of Medical Sciences, Student’s Research Group “Biological Psychiatry”, Department of Psychiatric Genetics, Faculty of Medical Sciences, Poznan University of Medical Sciences, 60-806 Poznan, Poland; 2 Department of Neural Engineering and Space Medicine, Collegium Medicum, University of Zielona Gora, 65-417 Zielona Gora, Poland; 3 Center for Children and Adolescent Treatment in Zabor, 66-003 Zabor, Poland; 4 Independent Researcher, 60-806 Poznan, Poland; 5 Department of Psychiatric Genetics, Faculty of Medical Sciences, Poznan University of Medical Sciences, 61-701 Poznan, Poland

**Keywords:** coping styles, defense mechanisms, brain-derived neurotrophic factor (BDNF), tissue plasminogen activator (tPA), matrix metalloproteinase 9 (MMP-9), Defense Style Questionnaire (DSQ-40), Coping Orientation to Problems Experienced Inventory (COPE)

## Abstract

Background/Objectives: An increasing number of studies are exploring how stress influences the development of various psychiatric and physical disorders. Psychological coping strategies and defense mechanisms play a vital role in managing stress. However, the biological mechanisms involved in coping with stress have not been thoroughly researched. This study focuses on the relationships between plasma levels of tPA-BDNF pathway proteins and their correlations with coping strategies and defense mechanisms. Methods: The study involved 48 healthy young adults. All participants completed the self-reported Defense Style Questionnaire (DSQ-40) and Coping Orientation to Problems Experienced Inventory (COPE). BDNF, proBDNF, t-plasminogen activator/tPA, total serpin E1/PAI-1, serpin F2/alpha 2-antiplasmin, and MMP-9 plasma concentrations were determined using ELISA. Results: We detected higher BDNF and lower MMP-9 levels in females. We found differences in the DSQ-40 humor subdimension and in the COPE focus on and venting of emotions category between women and men. We found correlations between studied protein plasma concentrations. Positive correlations of total serpin E1/PAI-1 with denial and mental disengagement and negative correlations with some active coping categories were found. Correlations of DSQ-40 scores with BDNF, proBDNF, MMP-9, and total serpin E1/PAI-1 were detected. Conclusions: Our findings indicate that there are functional associations between the proteins we studied and various coping styles, as well as mature, immature, and neurotic defense mechanisms.

## 1. Introduction

An increasing number of studies are investigating the impact of stress on the manifestation of various psychiatric and somatic disorders. An essential mediator between an objective level of stress and its influence on the risk of developing those diseases is how we cope with it [1,2]. Coping is the effort made to manage internal and external factors perceived as taxing or exceeding the person’s resources. It is used to reduce tension and restore psychological equilibrium [3]. Coping mechanisms can be categorized in many ways, such as mature, immature, active, and passive [4,5]. The functional strategies we use can positively impact how we manage stress and increase our resilience. On the other hand, the dysfunctional ones may exacerbate the harmful biological and psychological effects of stress. The use of dysfunctional ones is correlated with various psychiatric illnesses [1,6,7] and suicidal behaviors [8,9,10].

Research has demonstrated that patients with depression exhibit significant differences in coping mechanisms when compared to healthy individuals. They tend to utilize more avoidant strategies—such as denial, mental disengagement, and behavioral disengagement—and fewer problem-focused strategies, which include active coping, planning, suppression of competing activities, and positive reinterpretation [11]. Patients with bipolar disorder (BD) particularly show a significantly higher tendency to use behavioral disengagement and religious coping [12]. Coping mechanisms such as self-distraction and self-blame have been correlated with suicidal behaviors [13]. A study conducted on military personnel found a connection between maladaptive coping strategies—such as denial, behavioral disengagement, substance use, and self-blame—and suicidal ideation [14]. Moreover, avoidant coping mechanisms, including denial, behavioral disengagement, and mental disengagement, may be associated with an increased suicide risk among psychiatric patients [15]. A meta-analysis highlighted a correlation between eight specific coping subscales—particularly denial, behavioral disengagement, and mental disengagement—and heightened levels of distress, which manifests as depressive symptoms, anxiety, negative affect, general distress, and physical symptoms [16].

Brain-derived neurotrophic factor (BDNF) plays a crucial role in regulating activity-dependent neuroplasticity. Its concentration may be reduced in conditions such as neurodegenerative and neuropsychiatric diseases, where neuroplasticity processes are compromised [17,18,19]. Research has demonstrated that in cellular models of memory and learning—widely recognized as indicators of neuroplasticity—electrical stimulation that induces long-term potentiation (LTP) leads to an increase in BDNF expression [20]. The influence of BDNF has also been observed in mood-related behaviors and cognitive functions, reinforcing its significance in the context of neuropsychiatric and neurodegenerative disorders [21]. Conditions such as major depressive disorder, bipolar disorder, eating disorders, addiction, schizophrenia, Parkinson’s disease, and Alzheimer’s disease exemplify this interest in BDNF [18,22,23].

The first product of BDNF synthesis is the precursor protein preproBDNF, which undergoes proteolytic cleavage with the participation of protein convertases to proBDNF and then to the final product, mature BDNF (mBDNF). Intracellular proteases are mainly responsible for proteolytic cleavage in trans-Golgi networks or secretory granules, including the prohormone proconvertases PC1, PC2, PC7, and furin. Nevertheless, extracellular proteases such as plasmin and matrix metalloproteinases (MMPs) also play an essential role after BDNF secretion in immature form [17,24,25]. The plasminogen–plasmin pathway is the main way of controlling fibrinolysis, and its inhibitors play a crucial role in sustaining an organism’s homeostasis. This is mainly achieved in two ways: by inhibiting the conversion of plasminogen into plasmin and by inhibiting plasmin. Tissue-type plasminogen activator (tPA) is a crucial protein that activates plasminogen into plasmin. It has been demonstrated that tPA plays a role in regulating the conversion of proBDNF into mBDNF through the activation of plasminogen [26,27]. Plasminogen activator inhibitor type 1 (PAI-1) is considered to be the main physiological inhibitor of plasminogen activation [28]. It is a member of the serpin (serine protease inhibitor) superfamily [29]. It binds the free tPA in plasma as well as tPA on the surface of endothelial cells and forms a tPA–PAI-1 complex that inactivates it [30]. Alpha-2 antiplasmin is the primary physiological inhibitor of plasmin and has been shown to inhibit the conversion of proBDNF into mBDNF [31].

Matrix metalloproteinases are a family of zinc-dependent endopeptidases that have been associated mainly with remodeling the extracellular matrix during processes such as embryogenesis, morphogenesis, angiogenesis, and others [32]. All metalloproteinases are synthesized as preproenzymes, with some of them, like MMP9, being secreted from cells as proenzymes. Their activity is tightly controlled in many ways, such as during their synthesis, where they can be induced by various factors, e.g., BDNF [33,34] and by being activated through mechanisms like other MMPs and the plasminogen–plasmin cascade [35]. Metalloproteinases contribute to neuropathology in various mechanisms, but they also play an important role in CNS development and repair [36]. MMP9 has been linked with synaptic plasticity [37] as well as with bipolar disorder [38] and depression [39,40].

ProBDNF, the substrate of the aforementioned proteases, is also a functional protein whose activity in the central nervous system is independent and sometimes even opposite to mBDNF [17]. The differences in their actions are already expressed at the level of interaction with cellular receptors. While mBDNF signals through its high-affinity tropomyosin-related kinase B (TrkB) receptor, proBDNF signals through the low-affinity neurotrophin receptor p75 [17,41]. BDNF is responsible for the maintenance and survival of the neurons, and proBDNF exerts proapoptotic properties. Its other functions are impairment of synaptic transmission and plasticity and inhibition of synapse formation and dendritic arborization [24]. The forms of this activity-dependent synaptic plasticity are long-term potentiation (LTP) and long-term depression (LTD) which increase and decrease the strength of synaptic transmission, respectively [19]. While BDNF is responsible for the strength and maintenance of synaptic LTP, proBDNF enhances the opposite process—LTD [18]. The ratio of proBDNF to mature BDNF influences the resultant effect of this neurotrophin [41,42].

This study aimed to investigate the relationships between plasma levels of brain-derived neurotrophic factor (BDNF), its precursor proBDNF, and proteins involved in the extracellular cleavage of proBDNF: tissue-type plasminogen activator (tPA), total serpin E1/PAI-1, serpin F2/alpha 2-antiplasmin, and matrix metalloproteinase 9 (MMP-9). Additionally, we were looking for correlations between the analyzed proteins and the results from the Coping Orientation to Problems Experienced Inventory (COPE) and the Defensive Style Questionnaire (DSQ-40) in young, healthy adults.

## 2. Materials and Methods

### 2.1. Participants

The study involved 48 healthy young adults aged 19–31: *n* = 24 females, with a mean age of 23.7 (SD 2.8), and *n* = 24 males, with a mean age of 24.9 (SD 3.2). The majority of the participants were medical students (*n* = 33), and fifteen subjects had finished higher education and were employed. Exclusion criteria included any psychiatric diagnoses, having first-degree relatives with psychiatric disorders, severe somatic or neurological conditions, misuse of psychoactive substances, pregnancy, or breastfeeding. History of somatic and psychiatric diagnosis was established based on a self-report socio-demographic questionnaire [43]. All participants were of Caucasian descent and were recruited between May 2022 and December 2022 at the Department of Psychiatric Genetics at Poznan University of Medical Sciences in Poland.

All participants provided written informed consent. The study was conducted in accordance with the Helsinki Declaration, and the study protocol was approved by the Poznan University of Medical Sciences Ethics Committee (permission 39/22).

### 2.2. Defense Mechanisms and Coping Strategies

All participants completed the self-reported Defense Style Questionnaire (DSQ-40) [44] and Coping Orientation to Problems Experienced Inventory (COPE) [45]. The DSQ-40 assesses three different defense styles, represented by several defense mechanisms: mature defense style, which is composed of the subdimensions anticipation, humor, sublimation, and suppression; neurotic defense style, which consists of idealization, pseudo-altruism, reaction formation, and undoing; and immature defense style, which includes acting out, autistic fantasy, denial, devaluation, displacement, dissociation, isolation, passive aggression, projection, rationalization, splitting, and somatization [44]. The COPE was developed to assess a broad range of coping responses. It consists of 60 statements, which are then grouped into factors: positive reinterpretation and growth, mental disengagement, focus on and venting of emotions, use of instrumental social support, active coping, denial, religious coping, humor, behavioral disengagement, restraint, use of emotional social support, substance use, acceptance, suppression of competing activities, and planning [45].

### 2.3. Plasma BDNF, ProBDNF, t-Plasminogen Activator/tPA, Total Serpin E1/PAI-1, Serpin F2/Alpha 2-Antiplasmin, MMP-9 Quantification

Five milliliters of venous blood was drawn into BD Vacutainer^®^ PPT™ tubes, with K2EDTA as anticoagulant and gel separator (catalogue number 362795) between 07:30 and 09:30 after overnight fasting. Plasma was separated within an hour after blood was drawn using centrifugation (according to manufacturer’s instructions: 1100 RCF for 10 min), aliquoted, and stored at −80 °C until analyses.

BDNF, proBDNF, t-plasminogen activator/tPA, total serpin E1/PAI-1, serpin F2/alpha 2-antiplasmin, and MMP-9 plasma concentration quantification was performed using DuoSet ELISA kits (catalogue numbers DY248, DY3175, DY7449-05, DY9387-05, DY1470-05, DY911, respectively, R&D Systems, Minneapolis, MN, USA). ELISA was performed according to manufacturers’ instructions, with minor modifications described in details previously [46]. For each quantified protein, plasma dilution factor was determined prior to the analyses to fit the linear range of the standard curve. All samples and standards were run in duplicate, with intra-assay CV < 5% and inter-assay CV < 10%.

### 2.4. Statistical Analyses

Kolmogorov–Smirnoff and Lilliefors tests were used to check the normality of the data. Most of the studied clinical and biological variables showed non-normal distribution; thus, non-parametric methods were used. Mann–Whitney U-test and Spearman rank correlation was applied. The significance level was set at *p* < 0.05. In the correlation analysis, power of ≥80 with the study group of *n* = 48 was achieved with R ≥ 0.4 (https://sample-size.net/correlation-sample-size/, accessed on 25 May 2025). The statistical analyses were performed using Statistica v13 software (StatSoft, Krakow, Poland).

## 3. Results

### 3.1. Analysis of Plasma Levels of BDNF, ProBDNF, t-Plasminogen Activator/tPA, Total Serpin E1/PAI-1, Serpin F2/Alpha 2-Antiplasmin, and MMP-9 with Regard to Gender

Means of age and plasma levels of the studied proteins—BDNF, proBDNF, t-plasminogen activator/tPA, total serpin E1/PAI-1, serpin F2/alpha 2-antiplasmin, and MMP-9—are presented in Table 1. Our findings indicated that BDNF levels were higher (*p* = 0.04) and MMP-9 levels lower (*p* = 0.04) in females compared to males.

### 3.2. Analysis of Defense Style Questionnaire (DSQ-40) and Coping Orientation to Problems Experienced Inventory (COPE) Scores with Regard to Gender

**Figure 1:**
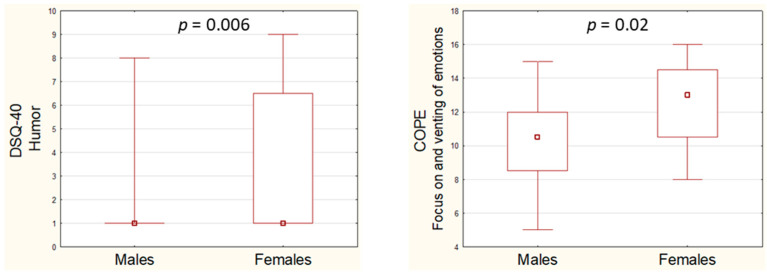
Analysis of Coping Orientation to Problems Experienced Inventory (COPE) and Defense Style Questionnaire (DSQ-40) scores with regard to gender.

We observed significantly higher scores on the DSQ-40 humor dimension (*p* = 0.006) and in the COPE focus on and venting of emotions category (*p* = 0.02) among females compared to males. No other differences were found in the analyses of the DSQ-40 or COPE results concerning gender. Results of the DSQ-40 and COPE with regard to gender are presented in supplementary Table S1. The significant results are presented in Figure 1.

### 3.3. Correlation Analysis of Plasma Concentrations of tPA-BDNF Pathway

**Figure 2:**
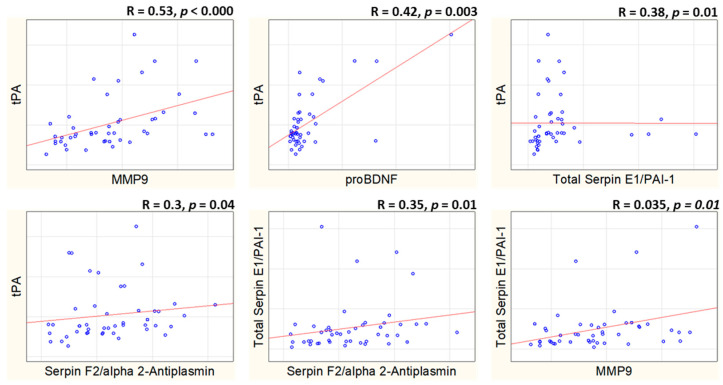
Correlations between tPA-BDNF activation pathway protein plasma concentrations.

We conducted a Spearman correlation analysis on the protein concentrations of the tPA-BDNF pathway. Our findings revealed moderate correlations between t-plasminogen activator (tPA) and several proteins: MMP-9 (*p* < 0.001), proBDNF (*p* = 0.003), total serpin E1/PAI-1 (*p* = 0.01), and serpin F2/alpha 2-antiplasmin (*p* = 0.04). However, no correlation was observed between plasma tPA and BDNF (*p* = 0.15). Additionally, total serpin E1/PAI-1 showed a correlation with serpin F2/alpha 2-antiplasmin (*p* = 0.01) and MMP-9 (*p* = 0.02). No other significant correlations among the studied proteins were identified. The significant results of the Spearman’s correlation analysis are presented in Figure 2.

### 3.4. Correlations of tPA-BDNF Pathway Plasma Protein Levels with Coping Orientation to Problems Experienced Inventory (COPE) and Defense Style Questionnaire (DSQ-40) Scores

We detected positive correlations between total serpin E1/PAI-1 and two coping strategies: denial (*p* = 0.002) and mental disengagement (*p* = 0.03). Additionally, we found negative correlations between total serpin E1/PAI-1 and both the use of emotional (*p* = 0.002) and instrumental (*p* = 0.04) social support, as well as focus on and venting of emotions (*p* = 0.03). Furthermore, MMP-9 showed a negative correlation with focus on and venting of emotions (*p* = 0.02).

We identified the following correlations between protein levels and DSQ-40 scores: a positive correlation between BDNF and humor (*p* = 0.03), as well as between MMP-9 and the rationalization subdimension (*p* = 0.03) and between proBDNF and the neurotic dimension. In contrast, proBDNF was negatively correlated with acting out (*p* = 0.04), while total serpin E1/PAI-1 showed a negative correlation with devaluation (*p* = 0.04). No other significant correlations between tPA-BDNF pathway proteins and COPE or DSQ-40 scores were detected. Significant correlations are presented in Table 2.

## 4. Discussion

The diagnosis of psychiatric disorders is prone to numerous errors, which arise not only from the biological and clinical heterogeneity but also from the diagnostic process itself. Although many biological markers have been proposed to help objectify and enhance diagnostic accuracy, none have been found to be both reliable and specific. It has been suggested that rather than relying on a single protein, examining the correlation between different proteins or the profiles of multiple proteins may be more effective for this purpose [47]. While still not clinically validated, brain-derived neurotrophic factor (BDNF) appears to be a promising biomarker for the early diagnosis of various psychiatric disorders, as well as conditions that may predispose individuals to them. Reduced levels of brain-derived neurotrophic factor (BDNF) have been observed in various disorders [18], alongside reports of maladaptive coping strategies in response to stress [48,49]. We aimed to investigate whether plasma levels of BDNF, proBDNF, and proteins involved in the conversion of proBDNF to mature BDNF (mBDNF) correlate with specific coping strategies and defense mechanisms in young, healthy adults. Additionally, we plan to extend our research to include psychiatric populations. An imbalance between proBDNF and BDNF may predispose individuals to neuropsychiatric disorders, potentially due to an impaired response to stress. To the best of our knowledge, this is the first time such a correlation has been explored.

We detected gender differences in the concentrations of the proteins studied: higher concentrations of BDNF in women, which is consistent with previous findings [50], and MMP-9 in men, although most previous studies show no such correlation [51,52]. Differences in coping strategies (focus on and venting of emotions) and defense mechanisms (humor) detected in our study group with regard to gender align with those reported previously, with higher scores in emotional coping in women [53,54].

A correlation was observed between tPA and other studied proteins, except mBDNF: proBDNF, total serpin E1/PAI-1, serpin F2/alpha 2-antiplasmin, and MMP-9. Total serpin E1/PAI-1 correlated with serpin F2/alpha 2-antiplasmin, and MMP-9 levels. Our results suggest coordinated regulation of the tPA-BDNF pathway [26,27,35].

Our results indicate involvement of tPA-BDNF in the regulation of coping strategies and defense mechanisms. Orzechowska et al. (2022) found that depressive patients more often use denial and mental or behavioral disengagement [11]. We found a positive correlation of total serpin E1/PAI-1 with denial and mental disengagement. In contrast, a negative correlation was found between the use of emotional and instrumental social support and focus on and venting of emotions, which are considered active coping strategies [55]. Denial and mental disengagement are considered maladaptive coping strategies, often associated with reduced cognitive flexibility and poorer long-term mental health outcomes [56]. Prolonged activation of the HPA axis resulting in an elevated cortisol response may lead to higher levels of total serpin E1/PAI-1. This mechanism has also been linked to the formation of PTSD-like memory formation [57]. Elevated peripheral PAI-1 has been observed in Alzheimer’s [58] and Parkinson’s [59] diseases and is associated with neuroplasticity processes [60]. Conversely, use of emotional or instrumental social support and focus on and venting of emotions, which are considered as adaptive coping strategies, actively engage individuals in addressing stress. In our study, we found that these adaptive coping strategies were correlated with lower plasma total serpin E1/PAI-1 levels. Effective coping strategies can help regulate the stress response through the regulation of the HPA axis [61,62], ultimately reducing inflammatory processes and total serpin E1/PAI-1 levels. Weak negative correlation of total serpin E1/PAI-1 with immature defense style “devaluation” was also found. This result contrasts with the other obtained results, possibly due to the small study group and its related limitations.

We found a positive correlation between BDNF (brain-derived neurotrophic factor) and the use of humor as a mature defense mechanism. Humor helps individuals reframe stressful situations, and can reduce the emotional impact of those situations. It activates the brain’s reward pathways [63], decreases the perception of stress [64], and lowers inflammatory processes [65]. However, there is a lack of published studies that specifically connect BDNF with the use of psychologically mature defense mechanisms. Obtained results regarding proBDNF are inconsistent, with a positive correlation with the neurotic dimension, and a negative correlation with acting out (immature subdimension). While proBDNF exerts the opposite effect to mature BDNF—negatively regulates neuronal remodeling, and synaptic transmission [41]—its positive correlation with maladaptive neurotic defense mechanisms might be explained by disturbed mechanisms of synaptic plasticity [66]. Its negative correlation with acting out needs further investigation.

In our study, MMP-9 was found to have a negative correlation with adaptive coping strategies (focus on and expressing emotions), while positively correlating with rationalization, which is considered a component of immature defense mechanisms. Our findings suggest that MMP-9 may play a role in maladaptive coping strategies. Research on the role of MMP-9 in this context is limited. Hüfner et al. (2015) demonstrated an increase in plasma levels of MMP-9 during a combination of persistent mental stress and acute physical stress [67]. Elevated MMP-9 levels have also been reported in patients with ovarian cancer and depression. Furthermore, recent stressful life events were associated with higher MMP-9, while greater social support correlated with decrease in MMP-9 levels [68]. Results from animal model studies revealed an increase in MMP-9 expression in the hippocampus during a chronic immobilization test [69]. Therefore, our findings of elevated MMP-9 levels associated with maladaptive coping align with previous research indicating that MMP-9 expression increases in response to stress.

Our results suggest associations of mature defense mechanisms and active coping with stress, with possible reduction in activation of the HPA axis and/or more efficient synaptic plasticity.

## 5. Limitations

Our study is preliminary and exploratory in nature, and the correlational findings presented herein warrant further validation through replication in larger, more diverse samples, including individuals with psychiatric disorders. The current study cohort was predominantly composed of medical students, a population known to experience elevated stress levels. Future research should incorporate both self-reported assessments and physiological and behavioral measures to provide more comprehensive and robust evidence. It is important to acknowledge the potential biases inherent in self-report measures. The preliminary results obtained in our study require further investigation using longitudinal designs and/or experimental approaches, including animal or in vitro models.

## 6. Conclusions

This paper is the first to describe the correlations between psychological coping strategies and defense mechanisms with the circulating levels of tPA-BDNF pathway proteins. Our findings suggest that there are functional associations between the studied proteins and various coping styles, as well as mature, immature, and neurotic defense mechanisms. The mechanisms underlying the regulation of these psychological processes may involve the control of synaptic plasticity through the balance of long-term potentiation (LTP) and long-term depression (LTD), which is influenced by the ratio of mature BDNF to proBDNF. This ratio depends on the effectiveness of extracellular BDNF processing. The main limitation of the study is the small sample. Thus, the results of this study should be interpreted carefully in a preliminary and proof-of-concept manner. Future studies should involve larger cohorts and more biological factors involved in the regulation of BDNF expression.

## Figures and Tables

**Table 1:** Comparison of age and plasma proteins: BDNF, proBDNF, t-plasminogen activator/tPA, total serpin E1/PAI-1, serpin F2/alpha 2-antiplasmin, and MMP-9 with regard to gender.

		Whole Group	Males	Females	
	*n*	Mean (SD)	Mean (SD)	Mean (SD)	*p* ^1^
Age	48	24 (3)	25 (3)	24 (3)	ns
BDNF (ng/mL)	48	4.55 (8.91)	2.61 (2.57)	6.5 (12.15)	0.04
ProBDNF (ng/mL)	48	1.99 (2.91)	1.96 (2.13)	2.01 (3.57)	ns
tPA (pg/mL)	47	832 (528)	918 (510)	750 (543)	ns
Total serpin E1/PAI-1 (ng/mL)	48	51.07 (59.1)	59.74 (61.21)	42.4 (56.86)	ns
Serpin F2/alpha 2-antiplasmin (mg/mL)	47	9.32 (5.38)	8.23 (4.77)	10.36 (5.80)	ns
MMP-9 (ng/mL)	46	201.36 (89.87)	224.34 (66.83)	180.3 (103.71)	0.04

^1^ Mann–Whitney U-test, ns—non significant, BDNF—brain-derived neurotrophic factor, tPA—t-plasminogen activator, MMP-9—matrix metalloproteinase 9.

**Table 2:** Correlations of Coping Orientation to Problems Experienced Inventory (COPE) and Defense Style Questionnaire (DSQ-40) scores with plasma levels of tPA-BDNF pathway.

	R	*p* ^1^
**COPE**		
Total serpin E1/PAI-1 and denial	0.43	0.002
Total serpin E1/PAI-1 and denial	0.30	0.03
Total serpin E1/PAI-1 and use of emotional social support	−0.44	0.002
Total serpin E1/PAI-1 and focus on and venting of emotions	−0.32	0.03
Total serpin E1/PAI-1 and use of instrumental social support	−0.3	0.04
MMP-9 and focus on and venting of emotions	−0.35	0.02
**DSQ-40**		
BDNF and humor	0.31	0.03
proBDNF and neurotic	0.29	0.05
proBDNF and acting out	−0.29	0.04
Total serpin E1/PAI-1 and devaluation	−0.29	0.04
MMP-9 and rationalization	0.32	0.03

^1^ Spearman’s correlation PAI-1—plasminogen activator inhibitor, BDNF—brain-derived neurotrophic factor, MMP-9—matrix metalloproteinase 9.

## Data Availability

The original contributions presented in the study are included in the article/Supplementary Material. Further inquiries can be directed to the corresponding author.

## Supplementary Materials

The following supporting information can be downloaded at: https://www.mdpi.com/article/10.3390/brainsci15060575/s1; Table S1: Analysis of Coping Orientation to Problems Experienced Inventory (COPE) and Defense Styles Questionaire (DSQ-40) scores with regard to gender.

## References

[1] Wiszniewski B., Liberska H. (2022). Styles of Coping with Stress among Healthy People and People with Diagnosis of Schizophrenia and Selected Personality Dimensions. Int. J. Environ. Res. Public Health.

[2] Palego L., Giannaccini G., Betti L. (2021). Neuroendocrine Response to Psychosocial Stressors, Inflammation Mediators and Brain-Periphery Pathways of Adaptation. Cent. Nerv. Syst. Agents Med. Chem..

[3] Folkman S., Gellman M.D., Turner J.R. (2013). Stress: Appraisal and Coping. Encyclopedia of Behavioral Medicine.

[4] Stanisławski K. (2019). The Coping Circumplex Model: An Integrative Model of the Structure of Coping With Stress. Front. Psychol..

[5] Skinner E.A., Edge K., Altman J., Sherwood H. (2003). Searching for the Structure of Coping: A Review and Critique of Category Systems for Classifying Ways of Coping. Psychol. Bull..

[6] Bianca C.-S.D., Ramona P.L., Ioana M.V. (2022). The Relationship between Coping Strategies and Life Quality in Major Depressed Patients. Egypt. J. Neurol. Psychiatry Neurosurg..

[7] Ered A., Gibson L.E., Maxwell S.D., Cooper S., Ellman L.M. (2017). Coping as a Mediator of Stress and Psychotic-like Experiences. Eur. Psychiatry.

[8] Josepho S.A., Plutchik R. (1994). Stress, Coping, and Suicide Risk in Psychiatric Inpatients. Suicide Life-Threat. Behav..

[9] Caredda M., Vescera L., Picardi A., Tarolla E., Pancheri C., Biondi M., Tondo L. (2024). Positive Psychological Functioning, Resilience and Styles of Coping as Buffers against Suicidal Behaviours. A Case-Control Study. J. Affect. Disord..

[10] Horesh N., Rolnick T., Iancu I., Dannon P., Lepkifker E., Apter A., Kotler M. (1996). Coping Styles and Suicide Risk. Acta Psychiatr. Scand..

[11] Orzechowska A., Bliźniewska-Kowalska K., Gałecki P., Szulc A., Płaza O., Su K.-P., Georgescu D., Gałecka M. (2022). Ways of Coping with Stress among Patients with Depressive Disorders. J. Clin. Med..

[12] Aksoy Poyraz C., Özdemir A., Çakir Şen C., Usta Sağlam N.G., Enginkaya S., Tomruk N. (2021). The Impact of Coping Strategies on Suicide Attempts and Suicidal Ideation in Bipolar Disorder. J. Nerv. Ment. Dis..

[13] Lew B., Huen J., Yu P., Yuan L., Wang D.-F., Ping F., Abu Talib M., Lester D., Jia C.-X. (2019). Associations between Depression, Anxiety, Stress, Hopelessness, Subjective Well-Being, Coping Styles and Suicide in Chinese University Students. PLoS ONE.

[14] Khazem L.R., Law K.C., Green B.A., Anestis M.D. (2015). Examining the Relationship between Coping Strategies and Suicidal Desire in a Sample of United States Military Personnel. Compr. Psychiatry.

[15] Ambrus L., Sunnqvist C., Asp M., Westling S., Westrin Å. (2020). Coping and Suicide Risk in High Risk Psychiatric Patients. J. Ment. Health.

[16] Kato T. (2015). Frequently Used Coping Scales: A Meta-Analysis. Stress Health.

[17] Bathina S., Das U.N. (2015). Brain-Derived Neurotrophic Factor and Its Clinical Implications. Arch. Med. Sci..

[18] Autry A.E., Monteggia L.M. (2012). Brain-Derived Neurotrophic Factor and Neuropsychiatric Disorders. Pharmacol. Rev..

[19] Mizui T., Ishikawa Y., Kumanogoh H., Lume M., Matsumoto T., Hara T., Yamawaki S., Takahashi M., Shiosaka S., Itami C. (2015). BDNF Pro-Peptide Actions Facilitate Hippocampal LTD and Are Altered by the Common BDNF Polymorphism Val66Met. Proc. Natl. Acad. Sci. USA.

[20] Patterson S.L., Grover L.M., Schwartzkroin P.A., Bothwell M. (1992). Neurotrophin Expression in Rat Hippocampal Slices: A Stimulus Paradigm Inducing LTP in CA1 Evokes Increases in BDNF and NT-3 mRNAs. Neuron.

[21] Fan Y., Luan X., Wang X., Li H., Zhao H., Li S., Li X., Qiu Z. (2025). Exploring the Association between BDNF Related Signaling Pathways and Depression: A Literature Review. Brain Res. Bull..

[22] Gupta A.K., Gupta S., Mehan S., Khan Z., Das Gupta G., Narula A.S. (2025). Exploring the Connection Between BDNF/TrkB and AC/cAMP/PKA/CREB Signaling Pathways: Potential for Neuroprotection and Therapeutic Targets for Neurological Disorders. Mol. Neurobiol..

[23] Hayat M.R., Umair M., Ikhtiar H., Wazir S., Palwasha A., Shah M. (2024). The Relationship Between Brain-Derived Neurotrophic Factor and Serotonin in Major Depressive and Bipolar Disorders: A Cross-Sectional Analysis. Cureus.

[24] Arévalo J.C., Deogracias R. (2023). Mechanisms Controlling the Expression and Secretion of BDNF. Biomolecules.

[25] De Vincenti A.P., Ríos A.S., Paratcha G., Ledda F. (2019). Mechanisms That Modulate and Diversify BDNF Functions: Implications for Hippocampal Synaptic Plasticity. Front. Cell. Neurosci..

[26] Pang P.T., Teng H.K., Zaitsev E., Woo N.T., Sakata K., Zhen S., Teng K.K., Yung W.-H., Hempstead B.L., Lu B. (2004). Cleavage of proBDNF by tPA/Plasmin Is Essential for Long-Term Hippocampal Plasticity. Science.

[27] Lee R., Kermani P., Teng K.K., Hempstead B.L. (2001). Regulation of Cell Survival by Secreted Proneurotrophins. Science.

[28] Cesarman-Maus G., Hajjar K.A. (2005). Molecular Mechanisms of Fibrinolysis. Br. J. Haematol..

[29] Sprengers E.D., Kluft C. (1987). Plasminogen Activator Inhibitors. Blood.

[30] Urano T., Suzuki Y., Iwaki T., Sano H., Honkura N., Castellino F.J. (2019). Recognition of Plasminogen Activator Inhibitor Type 1 as the Primary Regulator of Fibrinolysis. Curr. Drug Targets.

[31] Mou X., Peterson C.B., Prosser R.A. (2009). Tissue-Type Plasminogen Activator-Plasmin-BDNF Modulate Glutamate-Induced Phase-Shifts of the Mouse Suprachiasmatic Circadian Clock in Vitro. Eur. J. Neurosci..

[32] Cabral-Pacheco G.A., Garza-Veloz I., Castruita-De la Rosa C., Ramirez-Acuña J.M., Perez-Romero B.A., Guerrero-Rodriguez J.F., Martinez-Avila N., Martinez-Fierro M.L. (2020). The Roles of Matrix Metalloproteinases and Their Inhibitors in Human Diseases. Int. J. Mol. Sci..

[33] Kuzniewska B., Rejmak E., Malik A.R., Jaworski J., Kaczmarek L., Kalita K. (2013). Brain-Derived Neurotrophic Factor Induces Matrix Metalloproteinase 9 Expression in Neurons via the Serum Response Factor/c-Fos Pathway. Mol. Cell. Biol..

[34] Zhang L., Hu Y., Sun C.-Y., Huang J., Chu Z.-B. (2008). Brain-derived neurotrophic factor promotes the secretion of MMP-9 in human myeloma cell through modulation of nucleus factor-kappaB. Zhonghua Xue Ye Xue Za Zhi.

[35] Nagase H. (1997). Activation Mechanisms of Matrix Metalloproteinases. Biol. Chem..

[36] Yong V.W., Power C., Forsyth P., Edwards D.R. (2001). Metalloproteinases in Biology and Pathology of the Nervous System. Nat. Rev. Neurosci..

[37] Dziembowska M., Wlodarczyk J. (2012). MMP9: A Novel Function in Synaptic Plasticity. Int. J. Biochem. Cell Biol..

[38] Rybakowski J.K., Remlinger-Molenda A., Czech-Kucharska A., Wojcicka M., Michalak M., Losy J. (2013). Increased Serum Matrix Metalloproteinase-9 (MMP-9) Levels in Young Patients during Bipolar Depression. J. Affect. Disord..

[39] Li H., Sheng Z., Khan S., Zhang R., Liu Y., Zhang Y., Yong V.W., Xue M. (2022). Matrix Metalloproteinase-9 as an Important Contributor to the Pathophysiology of Depression. Front. Neurol..

[40] Bobińska K., Szemraj J., Czarny P., Gałecki P. (2016). Expression and Activity of Metalloproteinases in Depression. Med. Sci. Monit..

[41] Yang J., Harte-Hargrove L.C., Siao C.-J., Marinic T., Clarke R., Ma Q., Jing D., LaFrancois J.J., Bath K.G., Mark W. (2014). proBDNF Negatively Regulates Neuronal Remodeling, Synaptic Transmission, and Synaptic Plasticity in Hippocampus. Cell Rep..

[42] Cao W., Duan J., Wang X., Zhong X., Hu Z., Huang F., Wang H., Zhang J., Li F., Zhang J. (2014). Early Enriched Environment Induces an Increased Conversion of proBDNF to BDNF in the Adult Rat’s Hippocampus. Behav. Brain Res..

[43] Gawęda Ł., Prochwicz K., Adamczyk P., Frydecka D., Misiak B., Kotowicz K., Szczepanowski R., Florkowski M., Nelson B. (2018). The Role of Self-Disturbances and Cognitive Biases in the Relationship between Traumatic Life Events and Psychosis Proneness in a Non-Clinical Sample. Schizophr. Res..

[44] Andrews G., Singh M., Bond M. (1993). The Defense Style Questionnaire. J. Nerv. Ment. Dis..

[45] Carver C., Scheier M., Weintraub J. (1989). Assessing Coping Strategies: A Theoretically Based Approach. J. Personal. Soc. Psychol..

[46] Skibinska M., Kapelski P., Dmitrzak-Weglarz M., Lepczynska N., Pawlak J., Twarowska-Hauser J., Szczepankiewicz A., Rajewska-Rager A. (2021). Elevated Epidermal Growth Factor (EGF) as Candidate Biomarker of Mood Disorders—Longitudinal Study in Adolescent and Young Adult Patients. J. Clin. Med..

[47] Chen S., Jiang H., Liu Y., Hou Z., Yue Y., Zhang Y., Zhao F., Xu Z., Li Y., Mou X. (2017). Combined Serum Levels of Multiple Proteins in tPA-BDNF Pathway May Aid the Diagnosis of Five Mental Disorders. Sci. Rep..

[48] Carvalho L.D.F., Reis A.M., Pianowski G. (2019). Investigating Correlations Between Defence Mechanisms and Pathological Personality Characteristics. Rev. Colomb. Psiquiatr..

[49] Rajewska-Rager A., Dmitrzak-Weglarz M., Lepczynska N., Kapelski P., Pawlak J., Skibinska M. (2023). Clinical Assessment of Impulsiveness and Defence Mechanisms in Young Patients with Mood Disorders in a Two-Year Prospective Study. Early Interv. Psychiatry.

[50] Lommatzsch M., Zingler D., Schuhbaeck K., Schloetcke K., Zingler C., Schuff-Werner P., Virchow J.C. (2005). The Impact of Age, Weight and Gender on BDNF Levels in Human Platelets and Plasma. Neurobiol. Aging.

[51] Tsiknia A.A., Sundermann E.E., Reas E.T., Edland S.D., Brewer J.B., Galasko D., Banks S.J. (2022). Alzheimer’s Disease Neuroimaging Initiative Sex Differences in Alzheimer’s Disease: Plasma MMP-9 and Markers of Disease Severity. Alzheimer’s Res. Ther..

[52] Jonsson A., Hjalmarsson C., Falk P., Ivarsson M.-L. (2016). Levels of Matrix Metalloproteinases Differ in Plasma and Serum—Aspects Regarding Analysis of Biological Markers in Cancer. Br. J. Cancer.

[53] Kelly M.M., Tyrka A.R., Price L.H., Carpenter L.L. (2008). Sex Differences in the Use of Coping Strategies: Predictors of Anxiety and Depressive Symptoms. Depress. Anxiety.

[54] Matud M.P. (2004). Gender Differences in Stress and Coping Styles. Personal. Individ. Differ..

[55] Carver C.S., Michalos A.C. (2014). Active Coping. Encyclopedia of Quality of Life and Well-Being Research.

[56] Uchino B.N. (2006). Social Support and Health: A Review of Physiological Processes Potentially Underlying Links to Disease Outcomes. J. Behav. Med..

[57] Mennesson M., Revest J.-M. (2023). Glucocorticoid-Responsive Tissue Plasminogen Activator (tPA) and Its Inhibitor Plasminogen Activator Inhibitor-1 (PAI-1): Relevance in Stress-Related Psychiatric Disorders. Int. J. Mol. Sci..

[58] Angelucci F., Veverova K., Katonová A., Vyhnalek M., Hort J. (2023). Serum PAI-1/BDNF Ratio Is Increased in Alzheimer’s Disease and Correlates with Disease Severity. ACS Omega.

[59] Tanrikulu A.M., Ozdilek B., Agirbasli M. (2024). Serum Levels of Plasminogen Activator Inhibitor-1 in Patients with Parkinson’s Disease. Med. Princ. Pract..

[60] Stevenson T.K., Moore S.J., Murphy G.G., Lawrence D.A. (2022). Tissue Plasminogen Activator in Central Nervous System Physiology and Pathology: From Synaptic Plasticity to Alzheimer’s Disease. Semin. Thromb. Hemost..

[61] Roth M.K., Bingham B., Shah A., Joshi A., Frazer A., Strong R., Morilak D.A. (2012). Effects of Chronic plus Acute Prolonged Stress on Measures of Coping Style, Anxiety, and Evoked HPA-Axis Reactivity. Neuropharmacology.

[62] Radley J.J., Johnson S.B. (2018). Anteroventral Bed Nuclei of the Stria Terminalis Neurocircuitry: Towards an Integration of HPA Axis Modulation with Coping Behaviors—Curt Richter Award Paper 2017. Psychoneuroendocrinology.

[63] Mobbs D., Greicius M.D., Abdel-Azim E., Menon V., Reiss A.L. (2003). Humor Modulates the Mesolimbic Reward Centers. Neuron.

[64] Simione L., Gnagnarella C. (2023). Humor Coping Reduces the Positive Relationship between Avoidance Coping Strategies and Perceived Stress: A Moderation Analysis. Behav. Sci..

[65] Bennett M.P., Lengacher C. (2009). Humor and Laughter May Influence Health IV. Humor and Immune Function. Evid.-Based Complement. Altern. Med..

[66] Bowins B. (2004). Psychological Defense Mechanisms: A New Perspective. Am. J. Psychoanal..

[67] Hüfner K., Koudouovoh-Tripp P., Kandler C., Hochstrasser T., Malik P., Giesinger J., Semenitz B., Humpel C., Sperner-Unterweger B. (2015). Differential Changes in Platelet Reactivity Induced by Acute Physical Compared to Persistent Mental Stress. Physiol. Behav..

[68] Lutgendorf S.K., Lamkin D.M., Jennings N.B., Arevalo J.M.G., Penedo F., DeGeest K., Langley R.R., Lucci J.A., Cole S.W., Lubaroff D.M. (2008). Biobehavioral Influences on Matrix Metalloproteinase Expression in Ovarian Carcinoma. Clin. Cancer Res..

[69] van der Kooij M.A., Fantin M., Rejmak E., Grosse J., Zanoletti O., Fournier C., Ganguly K., Kalita K., Kaczmarek L., Sandi C. (2014). Role for MMP-9 in Stress-Induced Downregulation of Nectin-3 in Hippocampal CA1 and Associated Behavioural Alterations. Nat. Commun..

