# Supplementary material for: Coping Styles and Defense Mechanisms in Healthy Young Adults—Correlations with tPA-BDNF Pathway
Source: Brain Sci. 2025 May 26;15(6):575. doi: 10.3390/brainsci15060575 (PMC12190725; doi:10.3390/brainsci15060575)
Supplement: Supplementary file 1 [file brainsci-15-00575-s001.zip › brainsci-3628508-supplementary.pdf]

Supplementary Table 1. Analysis of Coping Orientation to Problems Experienced Inventory (COPE) and Defense Styles Questionaire (DSQ-40) scores with regard to gender.

	n	Whole group Mean (SD)	Males Mean (SD)	Females Mean (SD)	p[1]
		COPE			
Positive reinterpretation and growth	48	11.75 (2.03)	11.88 (1.75)	11.63 (2.30)	ns
Mental disengagement	48	8.79 (2.13)	8.42 (1.95)	9.17 (2.28)	ns
Focus on and venting of emotions	48	11.58 (2.74)	10.63 (2.57)	12.54 (2.62)	0.02
Use of instrumental social support	48	11.85 (3.11)	12.00 (3.36)	11.71 (2.91)	ns
Active coping	48	11.73 (1.63)	11.67 (1.88)	11.79 (1.38)	ns
Denial	48	6.10 (1.85)	6.21 (1.96)	6.00 (1.77)	ns
Religious coping	48	5.63 (2.65)	5.63 (2.55)	5.63 (2.79)	ns
Humor	48	7.88 (2.70)	8.00 (2.84)	7.75(2.61)	ns
Behavioral disengagement	48	6.69 (1.85)	6.71 (1.73)	6.67 (1.99)	ns
Restraint	48	10.15 (1.74)	9.96 (1.65)	10.33 (1.83)	ns
Use of emotional social support	48	11.40 (3.25)	11.00 (3.48)	11.79 (3.02)	ns
Substance use	48	5.81 (2.40)	5.96 (2.51)	5.67 (2.33)	ns
Acceptance	48	10.21 (2.69)	10.33 (2.39)	10.08 (3.01)	ns
Suppression of competing activities	48	10.46 (2.13)	10.58 (2.62)	10.33 (1.55)	ns
Planning	48	12.33 (2.06)	12.00 (2.38)	12.67 (1.66)	ns
		DSQ-40			
Sublimation	48	4.21 (3.36)	3.88 (3.29)	4.54 (3.48)	ns
Humour	48	2.31 (2.67)	1.29 (1.43)	3.33 (3.21)	0.006
Anticipation	48	1.94 (2.38)	1.96 (2.33)	1.92 (2.48)	ns
Suppression	48	2.96 (2.99)	2.42 (2.64)	3.50 (3.27)	ns
MATURE	48	11.42 (6.52)	9.54 (5.63)	13.29 (6.91)	ns
Undoing	48	3.42 (2.61)	3.21 (2.40)	3.63 (2.84)	ns
Pseudo-altruism	48	3.46 (3.46)	3.46 (3.35)	3.46 (3.64)	ns
Idealization	48	3.73 (2.44)	3.63 (2.50)	3.83 (2.43)	ns
Reaction formation	48	3.63 (3.13)	4.08 (3.17)	3.17 (3.09)	ns
NEUROTIC	48	14.23 (5.74)	14.38 (5.28)	14.08 (6.28)	ns
Projection	48	4.42 (2.44)	4.54 (2.30)	4.29 (2.61)	ns
Passive agression	48	4.00 (2.56)	3.88 (2.58)	4.13 (2.59)	ns
Acting out	48	3.71 (3.05)	3.29 (2.85)	4.13 (3.23)	ns
Isolation	48	3.60 (2.84)	4.13 (3.11)	3.08 (2.50)	ns
Devaluation	48	4.56 (2.89)	4.63 (2.79)	4.50 (3.04)	ns
Autistic fantasy	48	3.48 (2.51)	3.79 (2.59)	3.17 (2.44)	ns
Denial	48	4.96 (2.60)	4.79 (2.75)	5.13 (2.49)	ns
Displacement	48	4.27 (2.94)	4.38 (2.83)	4.17 (3.12)	ns
Dissociation	48	4.44 (2.62)	4.54 (2.81)	4.33 (2.48)	ns
Splitting	48	4.33 (2.27)	4.29 (2.16)	4.38 (2.43)	ns
Rationalization	48	2.33 (2.82)	2.50 (3.01)	2.17 (2.68)	ns
Somatization	48	3.98 (3.21)	3.33 (2.91)	4.63 (3.42)	ns
IMMATURE	48	48.08 (10.75)	48.08 (11.68)	48.08 (9.99)	ns
